# Both high fat and high carbohydrate diets impair vagus nerve signaling of satiety

**DOI:** 10.1038/s41598-021-89465-0

**Published:** 2021-05-17

**Authors:** Hailley Loper, Monique Leinen, Logan Bassoff, Jack Sample, Mario Romero-Ortega, Kenneth J. Gustafson, Dawn M. Taylor, Matthew A. Schiefer

**Affiliations:** 1 Malcom Randall VA Medical Center, Gainesville, FL USA; 2 Department of Applied Physiology and Kinesiology, University of Florida, Gainesville, FL USA; 3 Louis Stokes Cleveland VA Medical Center, Cleveland, OH USA; 4 College of Medicine & Life Sciences, University of Toledo, Toledo, OH USA; 5 Departments of Biomedical Engineering and Biomedical Sciences, University of Houston, Houston, TX USA; 6 Department of Biomedical Engineering, Case Western Reserve University, Cleveland, OH USA; 7 Department of Neurosciences, The Cleveland Clinic, Cleveland, OH USA

**Keywords:** Obesity, Preclinical research, Biomedical engineering, Systems analysis

## Abstract

Obesity remains prevalent in the US. One potential treatment is vagus nerve stimulation (VNS), which activates the sensory afferents innervating the stomach that convey stomach volume and establish satiety. However, current VNS approaches and stimulus optimization could benefit from additional understanding of the underlying neural response to stomach distension. In this study, obesity-prone Sprague Dawley rats consumed a standard, high-carbohydrate, or high-fat diet for several months, leading to diet-induced obesity in the latter two groups. Under anesthesia, the neural activity in the vagus nerve was recorded with a penetrating microelectrode array while the stomach was distended with an implanted balloon. Vagal tone during distension was compared to baseline tone prior to distension. Responses were strongly correlated with stomach distension, but the sensitivity to distension was significantly lower in animals that had been fed the nonstandard diets. The results indicate that both high fat and high carbohydrate diets impair vagus activity.

## Introduction

Obesity, defined as a body mass index (BMI) ≥ 30 kg/m^2^, is very prevalent in the US^1^. Beyond the healthcare crisis of obesity, the economic costs of obesity are also staggering. In a worsening trend, 31 states have obesity rates ≥ 30% and none are below 20%^2^. From 2008 to 2018, the U.S. saw a year-over-year increase in these high obesity rates of 23%. More than 30% of U.S. adults are obese and > 70% of Veteran service personnel are estimated to be overweight or obese^3^. In the U.S., nearly 10% of all deaths are obesity-related^4,5^. The Surgeon General noted that obesity is a top priority that, left unaddressed, poses a significant threat to military readiness and national security^6^. Obesity-related expenses are estimated to be 20% of annual U.S. healthcare expenditures^7^. Recognizing the adverse impact of obesity, the National Institutes of Health devotes more than $1.3B annually to obesity research^8^.

Diet and lifestyle changes can reduce excess body weight (EBW), but have attrition rates of 30–60%^9,10^. Surgery is an increasingly popular option. Gastric banding has the lowest mortality rate (0.1%) but highest long-term failure rate (> 50%)^11^. Roux-en-Y gastric bypass, biliopancreatic diversion, and sleeve gastrectomy have lower failure rates but higher mortality rates^11–13^. These techniques increase stomach wall stretch which contributes to a feeling of fullness with less food. These stretch signals, in addition to other neural inputs from the digestive system, lead to a reduction in food intake^14^**.** Despite the risks and costs, approximately 230,000 Americans undergo weight-loss surgery each year^15^.

The vagal nerves convey the degree of stomach fullness to the brain via afferent visceral fibers. The majority of vagal fibers are sensory afferents and nearly all abdominal afferents of the vagus nerve are unmyelinated, including stretch-sensitive mechanoreceptors in the walls of the stomach^16,17^. As the stomach wall stretches, e.g. during food intake, the discharge rate of stretch-sensitive mechanoreceptors increases approximately 20%^18–21^. Both stomach contents and chronic diet have been shown to alter the neural responses of mechanoreceptors and feeding behavior^22–27^. Vagal nerve stimulation (VNS) has been shown to promote reduced food intake, cause weight loss and reduce cravings and appetite in animals^18,28–34^ and humans^35–37^. Increasing vagal afferent activity is thought to induce weight loss by signaling stomach distension and producing satiety. Therefore, VNS has the potential to serve as a fully reversible, less-invasive, lower risk alternative to other surgical options. However, optimal stimulation parameters are not fully understood and successful VNS parameters are quite variable across these studies. Further, other studies have found no clear relationship between VNS and weight loss^38,39^. Furthermore, several side effects of VNS have been reported: headache, cramps, bloating, abdominal pain, belching, dyspepsia (indigestion), gastroesophageal reflux (heartburn), nausea, and vomiting^40–42^.

Vagal nerve transection (vagotomy) has been reported to reduce obesity^43^, but long-term follow-up suggests limited efficacy^44,45^ and other studies have shown that vagotomy inhibits the inhibition of food consumption following ingestion of certain carbohydrates or fatty acids^46^. Since vagotomy can also cause weight loss, it has been proposed that vagal nerve conduction block through high frequency stimulation (VBLOC) could be effective for regulating food intake. VBLOC systems use a 5 kHz stimulus and reportedly induced 17% weight loss that reached statistical significance compared to sham by 18 months post-implant^40,42,47,48^, although there was no significant difference in weight loss at the 12-month mark in an earlier report^40^. While VBLOC was initially FDA-approved, the agency determined that this therapy has not proven effective and the system is no longer available. Further, computer simulations raise doubt as to if VBLOC actually blocks the nerve, or, instead, if it blocks portions of the nerve while activating other portions^49^. The exact mechanism is not well understood but may involve blocking aberrant signals that otherwise promote hyperphagia^50^.

These recent reports highlight the need for VNS stimulus optimization to treat obesity effectively while minimizing side effects. The study described here increases our understanding of the underlying neural response to stomach distension and how that response is altered after exposure to different diets. A better understanding of the natural vagal nerve response to stomach volume and contents will facilitate the design of more beneficial VNS patterns to induce weight loss more consistently in the future.

## Methods

### Ethical approval

The Institutional Animal Care and Use Committee (IACUC) at the Louis Stokes Cleveland Veterans Affairs Medical Center (LSCVAMC) and later at the Malcom Randall Veterans Affairs Medical Center (MRVAMC) approved the use of obesity-prone Sprague Dawley (OP-SD) male rats in this study. Charles River Labs initially bred the rats, but they discontinued the animal model during the study. Subsequently, the animal research facility at the LSCVAMC established and maintained a breeding colony that was later transferred to the University of Florida. All experiments were performed in accordance with the relevant guidelines and regulations set forth by Public Health Service, the Animal Welfare Act, the Guide for Care and Use of Laboratory Animals, and ARRIVE.

### Rat husbandry

Rats were maintained on a standard 12:12 light:dark cycle. Water and food were provided ad libitum. Rats were fed one of three diets detailed in Table 1 (Envigo Teklad): a standard rat chow; a high fat, low carbohydrate diet (HF-LC); or a low fat, high carbohydrate diet (LF-HC).

**Table 1:** Diet types tested.

% of calories from	Diet type		
	Standard	HF-LC	LF-HC
Fat	18	60.3	10.5
Carbohydrate	58	21.4	69.1
Protein	24	18.3	20.5
Manufacturer product number	2018	TD.06414	TD.08806

### Animals

At approximately the same time in the morning, rats were anesthetized with isoflurane (~ 3% with O_2_ flow at 1000 mL/min). The weight, abdominal circumference, thoracic circumference, and nose-to-anus length were recorded. The BMI was calculated to determine individual obesity levels^51^ based on weight, measured on a calibrated scale, and body length (nose-to-anus). The BMI was calculated using the equation BMI = (body weight)/(body length)^2^. The abdomen and the neck were shaved and the skin was cleaned with 70% isopropyl alcohol.

**Figure 1:**
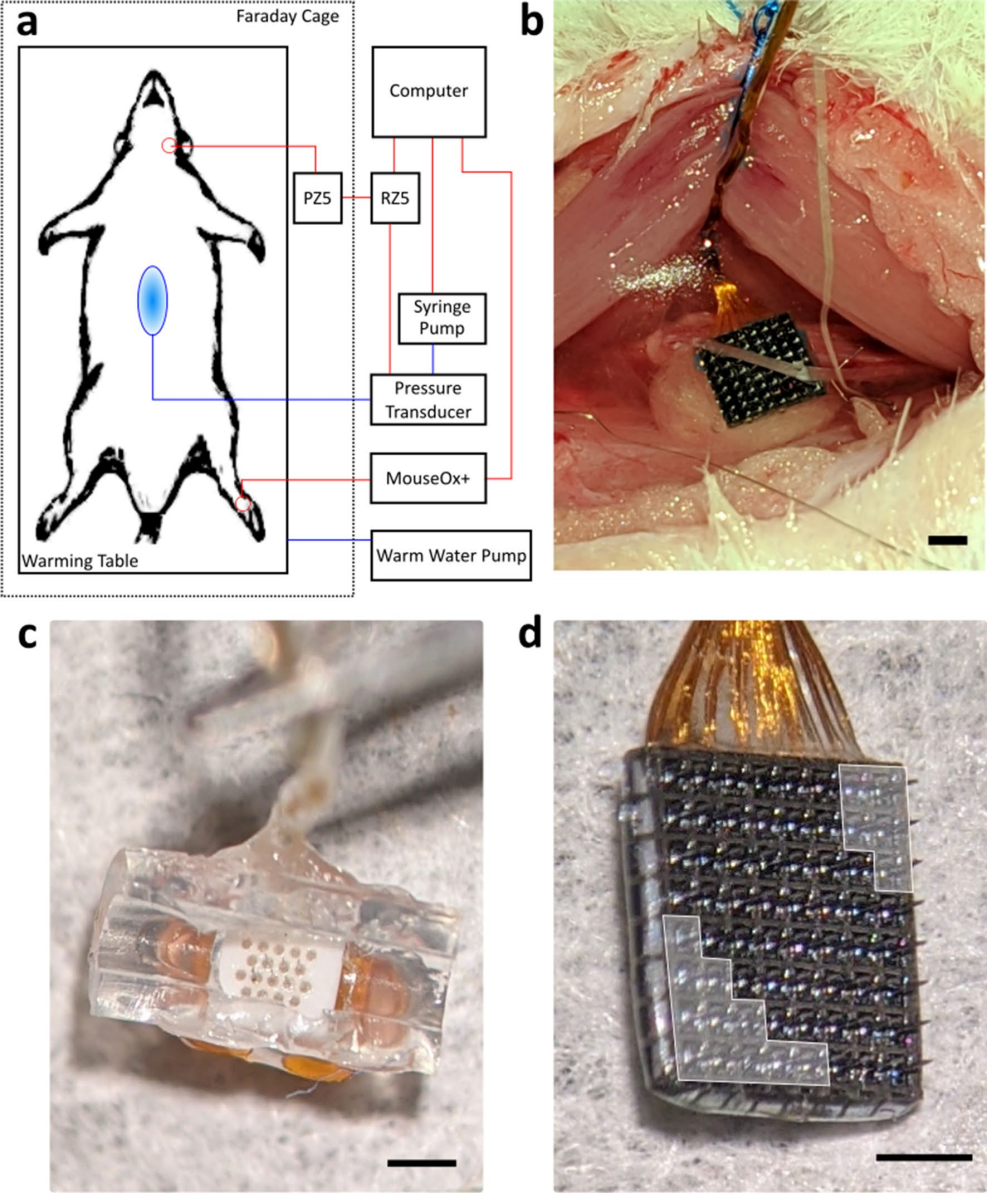
(**a**) Schematic of the experimental setup. Blue lines contain water. The blue oval represents the implanted balloon. Red lines are data lines. The red circle in the cervical area is the target implant site of the MEA. The red circle on the hind paw was the target site of the infrared vitals-monitoring clip. (**b**) View of the cervical vagus nerve implanted with the Blackrock MEA. (**c**) Microprobes MEA. (**d**) Blackrock MEA. Note, that while the Blackrock array appears as a standard 8 × 8, 64-channel MEA, the electrodes in the array were wired to three different 16-channel headstages. Thus, the 8 × 8 array was divided into three 16-channel subarrays that were in a diagonal (bottom left to upper right) orientation and 16 additional channels within the 8 × 8 array (shaded white) were nonfunctional. For (**b**) to (**d**), the black bar is 1 mm.

Animals were positioned supine within a Faraday cage on top of an acrylic heating pad (Fig. 1) that allowed maintaining body temperature by circulating warm water with a pump (Gaymar) placed outside the Faraday cage. The Faraday cage sat upon a pressurized, vibration suppressing floating air table (Kinetic Systems) that was electrically grounded. An infrared (IR) clip was attached to the rat’s hind foot and used to monitor heart rate (HR), breathing rate (BR), and blood oxygenation (SpO_2_) via a MouseOx Plus pulse oximeter (Starr Life Sciences) that was positioned outside of the Faraday cage. These vitals were saved into a time-stamped record at 1 Hz. Isoflurane anesthesia was continuously monitored throughout the experiment and saved into a time-stamped record every 15 min. At the conclusion of the experiment, the animal was euthanized with Euthasol (390 mg/ml pentobarbital sodium and 50 mg/ml phenytoin sodium; Virbac).

#### Gastric balloon implantation

A midline incision was carefully made along the linea alba in the abdomen down to the fascia. The abdominal cavity was opened to expose the stomach, which was carefully freed from connective tissue. Under a surgical microscope, an incision was made in the corpus of the stomach along the greater curvature avoiding visible blood vessels. This location was chosen because it has been shown to have a lower density of vagal afferents that may be disrupted by the incision^52^. Stomach contents were gently expressed using cotton-tipped applicators. Because each diet had a unique color (standard was brown, LF-HC was yellow, HF-LC was green), the researchers were not blinded to the specific diet the animal had been kept on. Once the stomach was empty, a urodynamic balloon catheter (Laborie, 9 Fr) was inserted into the stomach. Stomach size varied but was always shorter than the length of the balloon. To prevent balloon inflation outside the stomach, the neck of the balloon was constricted with surgical tape. Additionally, the tape served as an anchor point for sutures. The stomach was sutured closed and the catheter affixed in place to ensure consistent balloon positioning. Finally, the abdomen was covered with a sheet of thermoplastic (Parafilm) to prevent dehydration of the abdominal cavity. The other end of the catheter was attached to a calibrated pressure transducer (Utah Medical), which was attached to a programmable syringe pump (Lucca Technologies). Pressure inside the balloon was measured and saved at a rate of 305 Hz via a data acquisition channel in the RZ5 Bioamp Processor (Tucker Davis Technologies, TDT).

#### Electrode implantation

A midline incision was carefully made along the anterior portion of the neck, avoiding damage to underlying structures. The overlying salivary gland was carefully resected. The fascia was grasped, and the musculature was carefully divided along the midline, revealing the trachea. The lateral musculature was then elevated and retracted to reveal the left cervical carotid sheath. This allowed for clear identification of the left cervical vagus nerve that was then freed from the carotid sheath and artery by careful dissection under a surgical microscope. A custom penetrating microelectrode array (MEA) fabricated either by MicroProbes or Blackrock was inserted into the nerve (Fig. 1). MEAs from MicroProbes contained six recording electrodes ranging in length from 0.5 to 0.6 mm, an inter-electrode spacing of 250 µm, and an average impedance of 771 ± 242 kΩ. The MEA substrate was a comprised of polished alumina and medical-grade epoxy. The electrodes were a 70/30 platinum iridium (PtIr) alloy insulated with parylene-C. Wires coming from the MEA were gold, individually insulated with Terester 966 polyester enamel, bundled, and further insulated with medical-grade silicone. MEAs from Blackrock contained 64 electrodes, of which 48 were functional and wired to three 16-channel headstages. Thus, each 8 × 8 array contained three physically connected but electrically independent 16-channel subarrays. In almost every experiment, all 16 recording channels in the subarray of the 8 × 8 Blackrock array were inside the nerve. Only one 16-channel subarray within the 8 × 8 array was used at a time. Each electrode had a length of 0.5 mm, an inter-electrode spacing of 400 µm, and an average impedance of 355 ± 197 kΩ. The MEA substrate was silicon. The electrodes were platinum insulated with parylene-C. Wires coming from the MEA were gold, individually insulated with parylene-C, bundled, and then further insulated with medical-grade silicone. The reference electrode was either in intimate contact with the nerve (a secondary wire from the Blackrock MEA) or inside the nerve (one of the penetrating electrodes in the MicroProbes MEA). Due to the presence of the silicone cuff around the MicroProbes MEA, visual confirmation that all electrodes were in the nerve was not possible. It was possible to see the electrodes inside the nerve for the Blackrock array. If a channel was determined not to be inside the nerve, the data from it was not included in the analysis. The ground electrode was positioned in the tissue near the nerve. A small piece of cotton cut from the tip of a cotton-tipped applicator was placed between the artery and the back of the MEA to minimize any pulsatile motion artefact during recordings. The cervical cavity was filled with saline to ensure the nerve remained moist. The MEA was attached to a PZ5 amplifier/digitizer (Tucker Davis Technologies, TDT), which was connected to an RZ5 Bioamp Processer (TDT). The PZ5 had a 2× gain and an 18-bit A/D convertor that oversamples at 1 MHz with signal averaging to increase the bit depth. At the system sampling rate of 25 kHz, this produced a 0.15 µV resolution. The PZ5 also had an anti-aliasing FIR filter with a cutoff of approximately 11 kHz and a high pass filter at 0.1 Hz. Prior to the distension phase, the rat was not manipulated for 30 min to allow the system to stabilize. During this time, the isoflurane dosage was slowly lowered toward 1% in 0.25% increments while closely monitoring heart and breathing rate to ensure there was not more than a 10% increase in either. Once either heart rate or breathing rate started to increase, the isoflurane dosage was no longer decreased. If the heart rate or breathing rate increased more than 10%, the isoflurane dosage was increased in 0.25% increments until vitals stabilized.

### Stomach distensions

With the MEA in place and the rat stabilized, the experiment continued with controlled stomach distensions. Using custom built software in Matlab (Mathworks) and Synapse (TDT), the syringe pump (GenieTouch) delivered fixed-volume injections at regular intervals to distend the stomach. Each trial included multiple fixed-volume injections to achieve a total stomach volume. Typically, the stomach was distended to 5 mL, although in rats with very small or very large stomachs the maximum distension was reduced to 4 mL or increased to 6 mL, respectively. The distension step size was randomly varied from trial to trial and was set at 0.1 mL, 0.2 mL, 0.5 mL, or 1.0 mL. Thus, there were fifty 0.1 mL serial distensions to achieve a 5 mL total stomach distension. Prior to the first distension in a series, neural activity was recorded for 30 s to establish a pre-distension baseline. The first distension in the series occurred at 30 s. In almost every trial, each distension in the series was separated by 30 s. After the final maximum distension volume was reached, neural activity was recoded for an additional 30 s, after which the volume was withdrawn from the balloon and the rat was allowed to stabilize for several minutes prior to the next distension trial.

### Data processing

Post-hoc data analysis was conducted in Matlab on the University of Florida’s high performance computing cluster (HiPerGator) utilizing parallel processing on graphics processing units (GPUs). Neural recordings, animal vitals, anesthesia settings, and balloon pressure were imported into Matlab. The neural recordings were bandpass filtered between 150 and 5000 Hz. Rarely spurious artefacts arose and those time points were removed from analysis. To quantify the neural activity or “tone” within the vagal nerve the ENG was rectified and integrated using Matlab’s trapz function over sequential 5-s non-overlapping windows to determine the area under the curve (AUC), producing multiple measures at each balloon volume. Thus, “tone” within this study was a direct measure from the nerve and not obtained through quantification of heart rate variability as some other studies have. The percent change in the AUC compared to the mean AUC obtained prior to distension was calculated and used for statistical analyses. Thus, each animal’s AUC metric prior to each distension trial was normalized to zero and served as a control for the trial. This process accounted for any non-stationarity in neural activity throughout the testing session.

Neither the stomach size nor the balloon size were exactly the same across rats. As a result, a fixed volume distension in one animal may elicit a different degree of stomach wall stretch than in another animal. To account for this, data were classified as belonging to one of four epochs: pre-distension or small, medium, or large distensions. Assignment to an epoch was based on the pressure inside the balloon relative to steady-state pressure at the last distension. Small epochs had a pressure less than 25% of the final sustained pressure at maximum distension. Medium epochs had a pressure between 25 and 75% of the final sustained pressure. Large epochs had a pressure above 75% of the final sustained pressure. Prior to any distension, data were always categorized as pre-distension. AUC for each epoch was determined as the average of multiple 5-s AUCs that fell within the epoch. Thus, epoch AUC was normalized by the duration of the epoch to account for differences in epoch duration.

### Statistical analyses

All processed data were exported to MiniTab for statistical analyses. Data were analyzed with a general linear model (ANOVA) with fixed (diet type, distension step, volume, epoch) factors. ANOVA covariates included HR, BR, SpO_2_, balloon pressure, age, abdominal circumference, thoracic circumference, weight, BMI, electrode impedance, isoflurane dose. Second-order interactions between these covariates were also included in the model. Electrode manufacturer was pooled due to similarity in the obtained recordings. To reduce the number of terms in the model, we utilized MiniTab’s stepwise function to perform a backward elimination of terms with the elimination criteria of α = 0.05. The statistical analysis did not include the animal number because the age, diet, and distension order was randomized, nor did it include the electrode number within the MEA. An ANOVA was also conducted to determine if stomach volume affected HR, BR, or SpO_2_. Results were considered significant for p < 0.05. Values are reported as across-subject µ ± SEM within a particular diet type unless otherwise noted.

## Results

A total of 210 distension trials across 37 obesity-prone Sprague Dawley rats are reported. Rats were divided among standard (N = 11), LF-HC (N = 12), and HF-LC (N = 14) diets. Sample size was based on prior pilot studies. For the custom diets, rats were maintained on the LF-HC diet or HF-LC diet for an average of 259 and 269 days, respectively, which was not significantly different (p = 0.978). Combined, the experiments and additional control trials (see Supplement for details) generated approximately two terabytes of data.

### Experimental population and vitals

**Figure 2:**
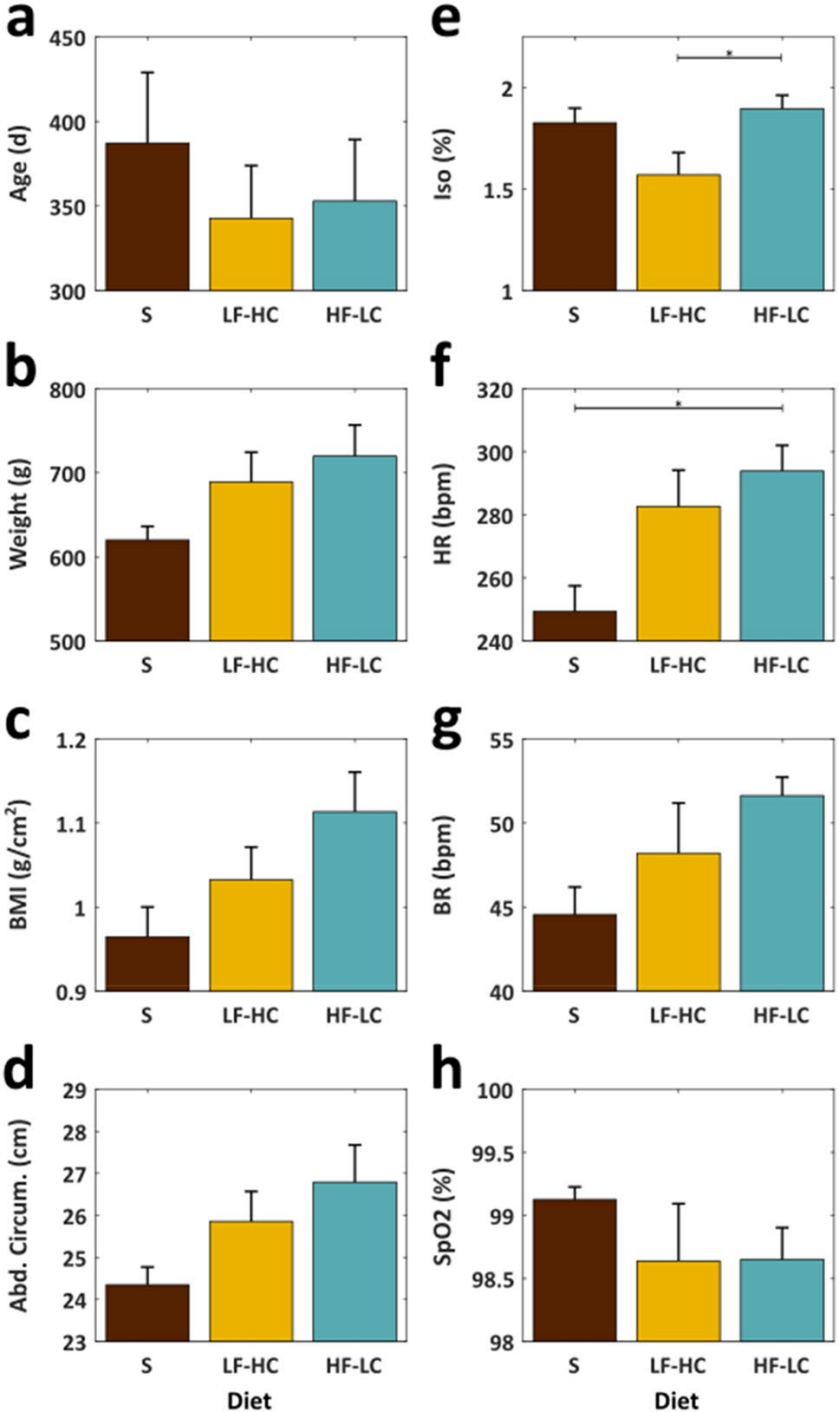
Various metrics for the animals, categorized by diet. Diet types are standard (S), low fat high carbohydrate (LF-HC) and high fat low carbohydrate (HF-LC). Significant differences (p ≤ 0.05) are denoted with “*”.

In terms of general health, rats on the standard diet tended to be healthier than those fed the HF-LC or LF-HC diets (see Fig. 2 and Supplementary Table 1). Rats on the HF-LC diet tended to be heavier and fatter with a higher heart rate and breathing rate and a lower blood oxygenation. Rats on the standard diet tended to be lighter, leaner, with a lower heart rate and breathing rate and a higher blood oxygenation. Rats on the LF-HC diet fell between these two groups.

Figure 2a–d shows age and body size metrics for the three groups at the time of surgery. Although none of the differences between groups reach statistical significance, rats on a nonstandard diet tended to be 9–14% heavier (p = 0.182), with a BMI that was 2–12% greater (p = 0.093) and an abdominal circumference that was 6–9% greater (p = 0.120) than those on a standard diet. Note the rats on the standard diet had a mean age that was slightly higher than the other two groups, which would typically be associated with that group also having *larger* body size metrics than groups with younger mean ages. However, that was not the case here providing further support that the near-significant trends seen in this study likely reflect real negative effects of these nonstandard diets on body size, weight, and fat composition.

Figure 2e–h shows the intraoperative isoflurane levels along with the resulting heart rate, breathing rate, and blood oxygenation for the three groups. Although isoflurane levels were similar between the standard diet and the HF-LC diet groups, the animals on the HF-LC diet had a mean heart rate that was 18% higher than those on a standard diet (p = 0.018) and a mean breathing rate that was 16% higher than those on a standard diet (p = 0.103). Accompanying these higher heart and breathing rates, rats on the HF-LC diet had a 0.5% lower blood oxygenation levels than those on a standard diet (p = 0.613). Animals fed the LF-HC diet exhibited similar trends but to a lesser extent (see Supplementary Table 1 for specific numbers for all metrics). Stomach distension did not significantly affect heart rate (p = 0.921), breathing rate (p = 0.972), SpO_2_ (p = 0.992), or the isoflurane dosage required (p = 0.988).

### Controls

In 28 rats and 117 trials, the vagal nerve was transected proximal to the recording MEA to remove any efferent contributions to the recorded signal. The percent change in AUC averaged across animals and MEA channels during distension was not significantly affected by the transection when compared against trials in which the nerve was intact (p = 0.893) (Supplementary Figure 1). Following transection, the diet and degree of distension remained significant factors (p = 0.017, p = 0.016, respectively). Because there was not a significant difference following transection, those datasets were included in the overall analysis.

Within the experimental design, each animal served as its own control because vagal tone was normalized by the baseline tone. Baseline tone was also analyzed using a secondary method. Using the mean and standard deviation of the multiple 5-s AUC values that quantified the baseline period, a 95% confidence interval in baseline tone was calculated, which then provided the amount of change in AUC that would be required to fall outside this confidence interval. The 95% confidence interval for the AUC prior to distension fell between − 0.08 and 0.11% (average of 0%). In the standard rats, all distension AUC values fell outside the baseline confidence interval. For rats on the other diets, distensions exceeding 1.5 mL resulted in AUCs outside the baseline confidence interval.

### Changes in recorded ENG as a function of distension

**Figure 3:**
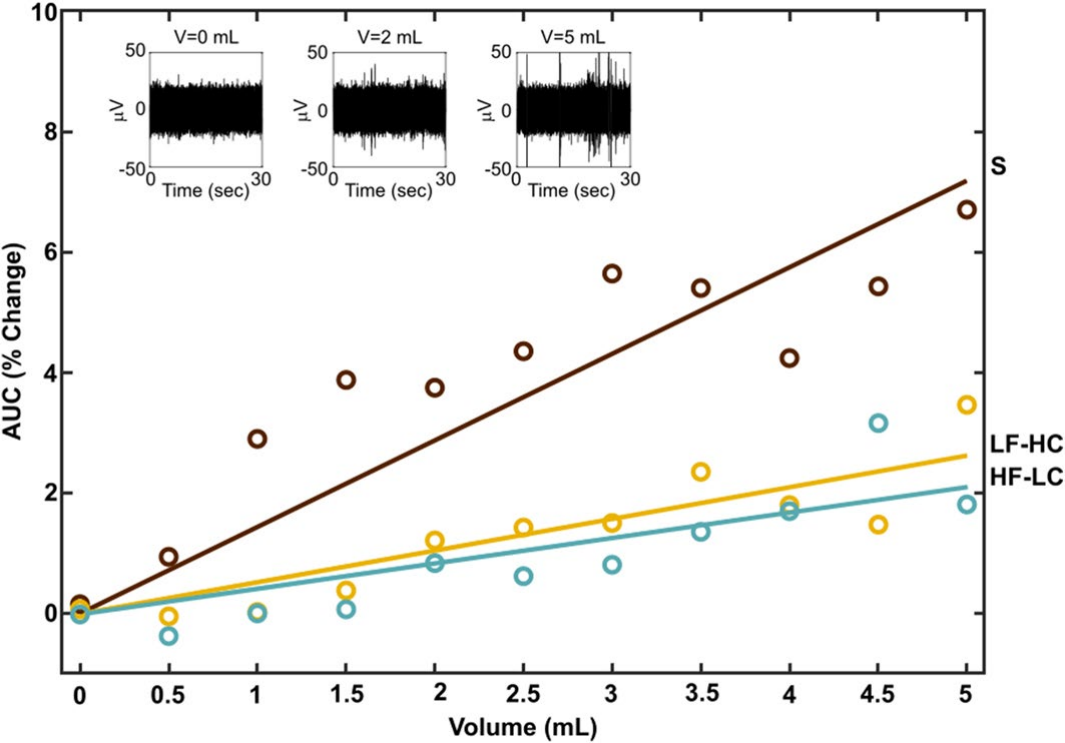
The change observed in the mean area under the curve (AUC) within vagal nerve recordings as a function of stomach volume across trials and MEA channels. Diet types are standard (S), low fat high carbohydrate (LF-HC), and high fat low carbohydrate (HF-LC). Circles indicate means across animals, time samples, and MEA channels for each injected volume binned in 0.5 mL increments. Linear regression R^2^ values: 0.70, 0.80, and 0.73 for the standard, LF-HC, and HF-LC diets, respectively. The insets show 30 s of raw ENG at three different volumes from one MEA channel of one trial in an animal fed the standard diet.

In 43% of experiments (16 out of 37), the MicroProbes array was used. Similarly, in 43% of experiments (16 out of 37), the Blackrock array was used. In 14% of experiments (5 out of 37), both arrays were used during portions of the experiment. Change in vagal nerve tone was quantified by rectifying and integrating the recorded ENG during stomach distension normalized against the tone recorded during baseline (non-distension). Across trials and MEA channels, change in vagal tone was strongly correlated with increasing stomach volume for all three diet types (Fig. 3) with a Pearson correlation ρ of 0.90 for each diet type. However, the sensitivity to stomach distension was significantly blunted (i.e., a lower slope) in animals that had been fed the HF-LC or LF-HC diets when compared to animals fed the standard diet (ANOVA p < 0.001). Animals fed the standard diet exhibited a 1.4% increase in AUC per milliliter of distension whereas animals fed the LF-HC or HF-LC diets exhibited a 0.5%/mL or 0.4%/mL increase, respectively. Depending on the volume of distension, the change in vagal tone in animals fed the LF-HC diet was less than 41% of the change in tone in animals fed the standard diet. The change in vagal tone in animals fed the HF-LC diet was similar to that recorded in animals fed the LF-HC diet. The distension step size, which was systematically varied across 0.1 mL, 0.2 mL, 0.5 mL, and 1.0 mL, was not found to have a significant effect (p = 0.575).

**Figure 4:**
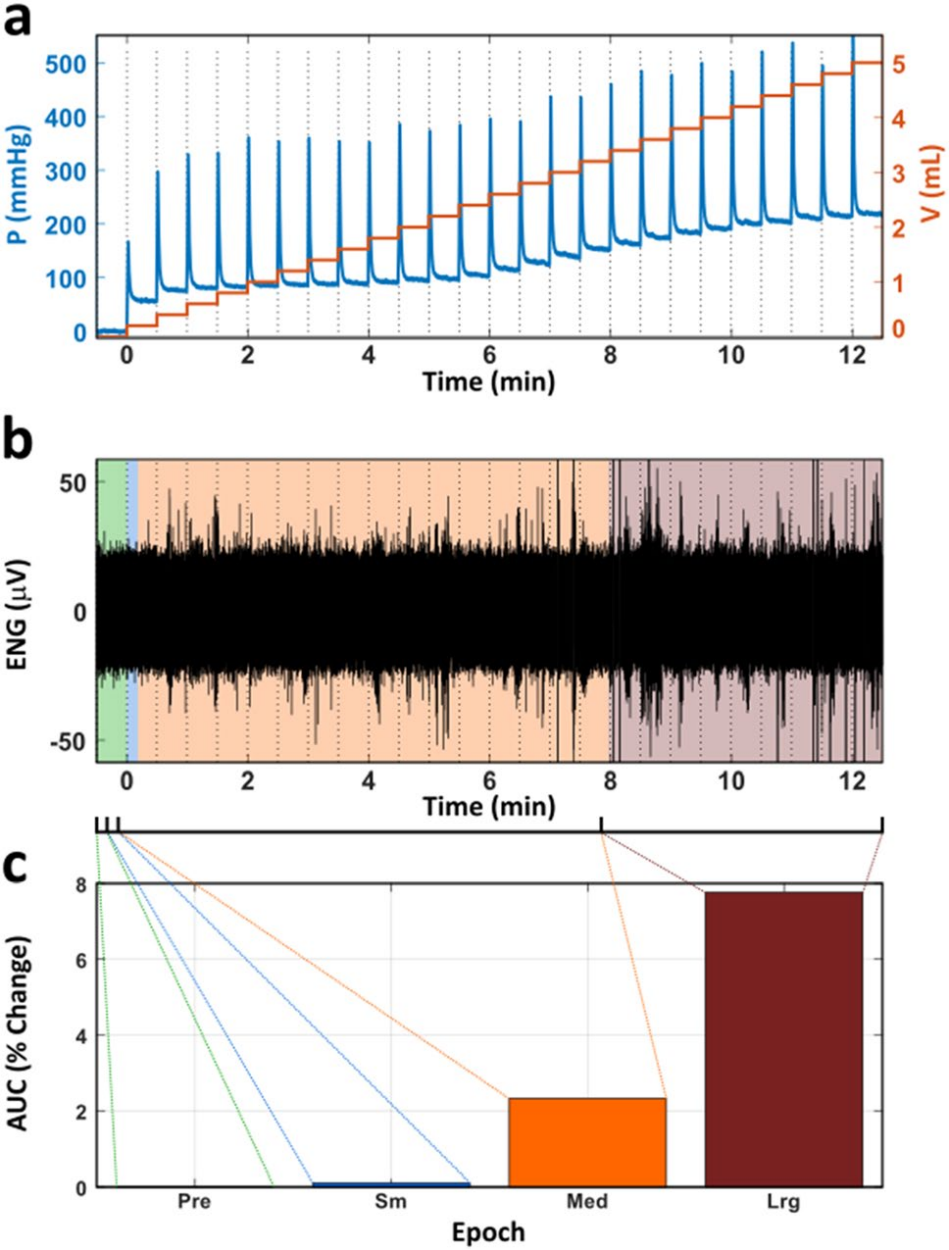
Example of defining dynamic epochs in one trial. (**a**) The pressure and injected volume in the intragastric balloon over the course of a trial. The distension step size was 0.2 mL. (**b**) The ENG recorded throughout the trial. (**c**) The percent change in AUC relative to pre-distension (baseline). Here, the bar height is the percent change in AUC averaged over every 5 s within the epoch (Pre: N = 30, Sm: N = 11, Med: N = 467, Lrg: N = 465). Data from animal on the standard diet.

Catheter pressure varied non-linearly with each step change in volume (Fig. 4a). Since activation of stomach stretch receptors is more directly related to pressure, we also compared groups when using pressure instead of volume to categorize distension epochs for statistical analysis. Specifically, the pressure relative to its final steady-state value at maximal distension was used to define the three post-distension epochs (Fig. 4b,c). Small (Sm) epochs exhibited pressures less than 25% of the maximum sustained pressure. Medium (Med) epochs exhibited pressures between 25 and 75% of the maximum sustained pressure. Large (Lrg) epochs exhibited pressures above 75% of the maximum sustained pressure. The AUC increased with increasingly large epochs (Fig. 4c). Examples from three additional animals are provided in the supplemental section.

**Figure 5:**
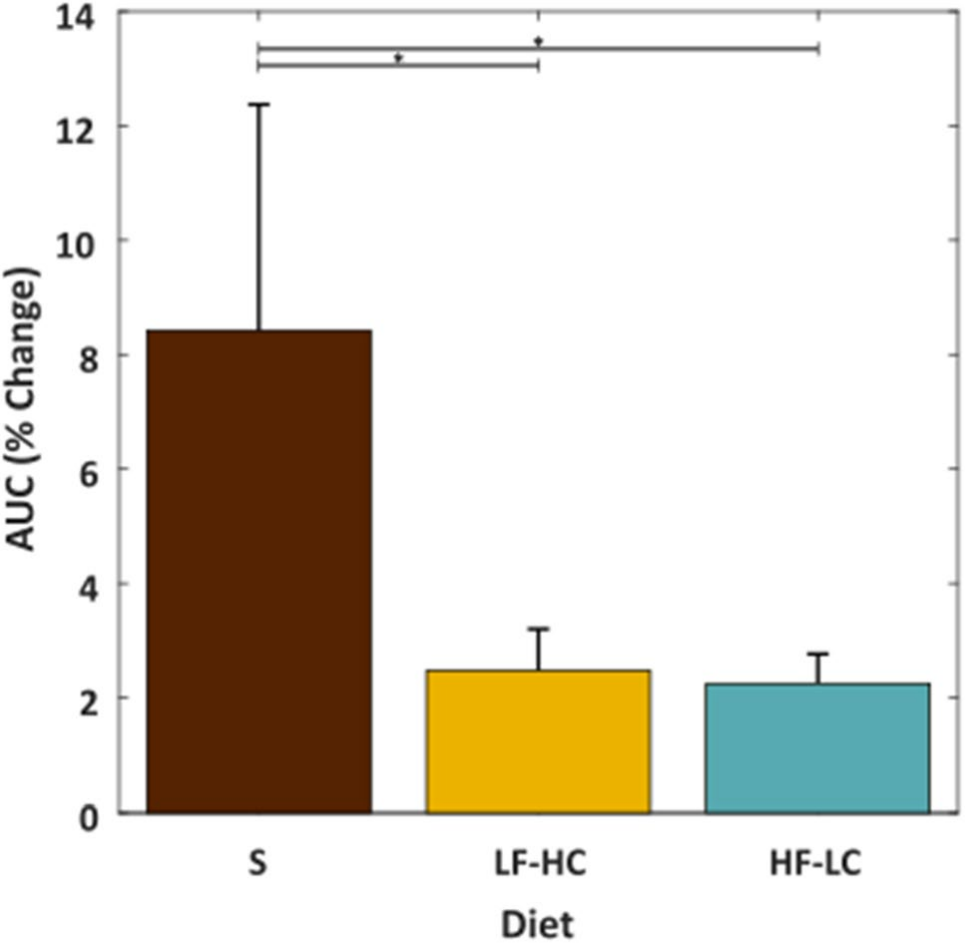
The change observed in the area under the curve (AUC) within vagal nerve recordings across animals, time samples, and MEA channels during large stomach distensions. Diet types are standard (S), low fat high carbohydrate (LF-HC), and high fat low carbohydrate (HF-LC). Significant differences (p ≤ 0.05) are denoted with “*”.

Because stomach distension is a primary driver of satiety^14,21^, we focused on the change in vagal tone during larger distensions that would play a significant role in cessation of eating. The change in vagal tone was significantly greater during larger distensions in animals that were fed the standard diet versus those that were fed either the LF-HC (p = 0.045) or HF-LC diet (p = 0.017) (Fig. 5). Weight (p = 0.863), BMI (p = 0.743), and abdominal circumference (p = 0.649) were not found to be significant factors.

## Discussion

### Effect of distension

Both diet type and stomach volume affected the AUC during distension. The percent increase in AUC over baseline was strongly and positively correlated with the distension volume. For any fixed distension, animals fed the standard diet exhibited a larger percent increase in AUC over baseline than animals fed the LF-HC or HF-LC diet. There was little difference between animals fed the LF-HC and HF-LC diets. Although there was an increase in vagal activity during stomach distension regardless of diet type, this study suggests that the increase in activity during stomach distention in animals on a nonstandard diet was only 20–25% of the increase observed in animals fed the standard diet. When focusing on larger distensions, which would be associated with having eaten a full meal, there was an average increase in AUC of 8.4% over baseline in rats fed the standard diet, which was 3×–4× larger than the 2.3–2.5% increase observed in rats on the HF-LC or LF-HC diets.

### Effect of diet

Gastric volume and the tension-sensitive receptors in the wall of the stomach are a strong influencer of afferent discharge and play a critical role in establishing satiety^14,21^ and reductions in the mechanosensitivity of these receptors contribute to hyperphagia. For example, mice lacking the transient receptor potential vanilloid 1 channel (TRPV1) have less sensitivity to stomach stretch and exhibit increased food intake^53,54^. Total loss of receptors, as would occur with subdiaphragmatic vagal deafferentation and vagotomy, causes overconsumption of palatable fluid in rats^46,55^. Vagal deafferentation of the gut contributes to decreased satiation in response to intragastric fat infusion^56^. Obese rodents fed a high fat diet also exhibit a reduction in mechanosensitivity and this may explain why obese animals consume more energy per meal^27^. Another study showed that a sustained high-calorie diet reduces vagal afferent sensitivity and expression of various receptors and neuropeptides^50^. Previous work showed that tension-sensitive gastric afferents exhibited up to approximately a 55% decrease in spikes per second when the animal had been fed a high fat diet^25,26,57^. A reduction in afferent sensitivity to jejunal stretch in animals fed a high fat diet has been reported^58^. The diminished AUC observed in animals fed the HF-LC diet is consistent with these prior studies and contributes to the growing body of evidence suggesting that animals fed a chronic high fat diet have a significantly decreased vagal response to stomach distension. Additionally, similar observations have been reported in rodents fed a high fat diet for shorter periods of time^23,59,60^. In fact, previous studies have shown that diets that were lower in fat than the HF-LC diet used here are also capable of altering expression of appetite hormone receptors^61^ and reducing vagally-mediated satiety signals^60^. Interestingly, “obesity-resistant” rats fed a high fat diet do not present with these conditions^61^.

Here, we show that obesity-prone rats fed the LF-HC diet exhibited similar trends to rats fed the HF-LC diet, and that both groups exhibited less change in vagal tone during distension than rats fed the standard diet with an intermediate amount of fat and carbohydrates. Although rats fed the HF-LC diet had a greater weight, BMI, and abdominal circumference than rats fed the LF-HC diet, the rats fed the LF-HC diet were more similar in these measures to those on the HF-LC diet than to those on the standard diet. Given that groups fed the HF-LC and LF-HC diets both exhibited lowered change in vagal tone, it suggests that the fat content within the diet is not solely responsible for the diminished response to stomach distension. While it is unclear if or how a high carbohydrate diet may lead to vagal afferent dysfunction, it has been shown that vagal afferents are important in both fat-induced and carbohydrate-induced satiation^55,62^. If a high carbohydrate diet leads to vagal afferent dysfunction similar to a high fat diet, then it would explain why both the HF-LC and LF-HC groups were similar in several metrics. Continued investigation into the underlying mechanisms including mechanosensor desensitization associated with both non-optimal diets as well as VNS is warranted.

### Controls

In this study, each rat served as its own control by normalizing post-distension AUC by pre-distension AUC. Further, the AUC prior to distension was analyzed to ensure that there was no effect due to time. In most trials, baseline ENG was recorded for 30 s. Additionally, there were control trials that had no distensions and “baseline” ENG was recorded for up to 250 s. In either scenario, the baseline AUC was not found to vary over time in any systematic way. Further, the AUC during distension fell outside of the 95% confidence interval of the baseline AUC. Proximal transection of the cervical vagal nerve between the MEA and the brain served as an additional control that allowed us to assess if efferent activity was a significant contributor to the recorded signal. A significant drop in tone would provide evidence that efferent fibers were at least partially responsible for the findings. The fact that transection had no significant effects indicates that any efferent signals provided minimal contributions to the recordings. These controls increase the confidence that the results observed were predominately afferent in nature, sensitive to both diet type and distension.

### Technical considerations

One challenge encountered in this study was the signal-to-noise ratio and the sensitivity of recordings to extraneous noise. To minimize the effects of extraneous noise, both electrical and mechanical, all recordings were conducted in a Faraday cage mounted on a pressurized floating air table. All electronic equipment was moved as far away from the Faraday cage as possible. The balloon catheter was secured with a magnetic alligator clip attached to the tabletop so that the balloon did not move relative to the rat. A small piece of cotton was positioned between the back of the MEA and the underlying carotid artery to reduce any mechanical motion associated with pulsating. These methods were essential because the overall size of the signal from naturally recruited, unmyelinated axons is very small. Nonetheless, there was variability in the recorded signals, some of which was undesirable, but some of which was typical of this system as illustrated in other studies^18–21^. Other sources of noise include muscle contraction, including heartbeat and breathing. These additional sources of noise could be filtered to some degree, but were not eliminated entirely during data processing. Although these sources were present, because the method of quantifying change in tone included normalization by baseline, and because these sources of noise were present also during baseline measures, their contribution to the quantified change in vagal tone were expected to be minimal. While neither heartrate nor breathing rate varied significantly with stomach distension, had either varied with distension the normalization method used here would not eliminate the effect of the noise source.

There is a risk for gradual changes in the baseline noise level that could lead one to conclude an increase in AUC when it really resulted from changes in the experimental preparation. Such drift and a correction for it was discussed in a recent publication^63^. The drift described by Davey et al. was not observed in this study perhaps because the nerve was left submerged to prevent dehydration or changes in impedance between the recording and reference electrodes. Further, had drift as described in Davey et al. occurred, it would be expected to affect all channels in the MEA as they were all closely spaced, but this was not observed.

A limitation in this study was the nature of the stomach distension. Balloon-based distension does not fully reflect the natural state of the post-prandial stomach and prior studies have shown that both the volume of the distension and the contents of the distending media are important and that stomach distension with nutritive contents elicits a more robust response^21,24^. While it would be more physiologically accurate if the stomach were filled with a fluid rather than a balloon, there are significant challenges to that approach. In early trials, we found that there was a significant risk of death due to backflow and aspiration of fluid. There was also a risk of fluid leaking around the catheter at the point it was inserted into the stomach. Due to leakage into the esophagus, leakage through the incision, movement of stomach contents into the small intestine, and absorption, direct infusion of fluid into the stomach may introduce too much variation into the study. Thus, while use of a balloon does not provide the full picture of the neural response to distension, it does allow us to focus solely on the response of mechanosensitive receptors without the confounding influence of chemosensitive receptors.

A potential confounding factor was mechanoreceptor adaptation, of which there were two sources. Because the animals had ad libitum access to chow prior to the experiment, it was possible that an animal started with a full stomach, but this was not always the case. If a stomach were full, then the mechanosensitive tension receptors may have adapted^64,65^. It is unlikely that this form of adaptation affected the results because there was no less than 1 h between the time that the stomach contents were expressed and the first recorded distension, providing ample time for the disadaptation of the receptors. However, the second source of adaptation—sustained stomach distension—persisted throughout the study. Also the recorded vagal afferents were likely a mix of ones that slowly and rapidly adapted to stomach distension^62^. While our results reflect the true physiological response, this mixed adaptation time course should be considered when interpreting the results. Peles et al.^18^ showed higher vagal afferent discharge when rapidly distending an empty stomach than when using multiple longer duration steps that gave enough time for the slowly adapting fibers to adjust. Additionally, hormones and stomach contents, among other factors, can modulate the vagal discharge to stomach stretch. For example, the response to distension can be modulated by circulating cholecystokinin (CCK) levels^62,66^. This adaptation occurs at the level of the stomach as evidenced by the loss of CCK influence on feeding following subdiaphragmatic vagotomy^67^. Conversely, ghrelin can significantly decrease vagal afferent activity^68^, as can leptin in lean-fed mice, but not in fasted or high fat fed obese mice^69^. Given the complexity of vagal response to stomach distension and the multiple factors that can modulate this response, the change in vagal tone reported here is best interpreted as the typical relative change in tone during a sustained distention in populations that predominately consumed specific types of foods (high fat, high carbohydrate, or mixed) rather than the maximum change in tone that could be achieved with the same volume of distension.

Isoflurane dosage may have introduced another source of variation. One study showed that isoflurane dose effected the number of spikes recorded between dosages of 1.5–2.0%^70^. In our study, isoflurane dosage ranged from 1.6 to 1.9% on average and a proper anesthesia level was determined per animal by closely monitoring HR, BR, and SpO_2_ to ensure these vitals fell within an expected range to indicate the animal was anesthetized deeply enough to not experience discomfort from the incision, distension, or paw pinch, but not so deep as to depress these vitals. The isoflurane concentration differed the most between the animals fed the LF-HC diet (1.6%) and those fed the HF-LC diet (1.9%), and this difference was marginally significant within the study. Interestingly, the percent change in AUC in these two groups was nearly identical for all levels of stomach distension and significantly less than the response recorded in animals fed the standard diet that averaged an isoflurane dosage of 1.8%. Since the nerve responses were lower in both the low (1.6%, LF-HC) and high (1.9%, HF-LC) anesthesia levels, compared to those at 1.8% (standard diet), it indicates that at this anesthesia range, the neural activity was not significantly affected, at least with regard to the natural response to stomach distension. While it is known that isoflurane concentration affects vagal tone, our results suggest that the effect of gastric distension on vagal tone can be determined while an animal is anesthetized with isoflurane.

There are two additional limitations of this study worth noting. First, the study was only performed on male rats. This was by design to reflect the older population of U.S. Veterans. To obtain data that is representative of the entire population, future studies should be conducted to include female rats. Second, recordings were only obtained from the left cervical vagus nerve. While the left cervical vagus nerve contains fibers from both the anterior and posterior gastric branches, more of the fibers come from the anterior branch^71^ and, as such, the contribution of the posterior gastric branch many not have been captured.

### Implications for VNS

We found that there was an approximately 2% increase in vagal tone above baseline during large distensions in rats on a nonstandard diet but a significantly higher 8% increase in rats on the standard diet. If the information conveyed by the vagus during these distensions is responsible for inducing satiety, then this study suggests that a VNS system would need to increase the change in vagal tone by approximately 4× in animals that have subsisted on a high fat or high carbohydrate diet in order to convey a large stomach distension. Certainly, suprathreshold stimulation could achieve an increase in vagal tone that exceeds this target, but it would come at the expense of battery life and may be excessive. It is foreseeable that a chronically implanted MEA could be used to monitor the change in vagal tone during food consumption and control a stimulator to augment fiber recruitment in a closed-loop fashion. Exactly how many or which vagal fibers need to be recruited during VNS to achieve this increase in tone is the focus of a future computational study which can also investigate the limitation of recording from a subset of fibers and the possible interactions between recording shank length and nerve diameter. Whether this trend holds true across different species has not yet been demonstrated.

Traditional experimental studies transect and divide the nerve until individual fibers that respond to stomach tension are found^62^. The benefit of transection and division is high signal fidelity. However, it does not represent a clinically-translatable approach. This study utilized penetrating MEAs to acquire the neural signal associated with distension. Penetrating MEAs have recently been used to monitor natural neural activity in the vagal nerve associated with breathing and changes in blood glucose in rats^72^ as well as ganglionic cell responses in the nodose ganglia during gastric distension in ferrets^73^. While the MEA does penetrate the nerve, the nerve remains intact. Thus, although the signal to noise ratio is unlikely to be as good as experimental methods that transect the nerve and tease out a fiber of interest, the approach has the potential for clinical deployment. Indeed, penetrating MEAs have been used in humans to restore tactile sensation^74,75^. Further, while single fiber recordings can provide incredibly insight, multi-unit recordings that provide more of an overall view are needed to understand better how future studies may best deliver stimulation to the nerve.

## Conclusions

This study found that the change in vagal tone was depressed significantly in animals fed either a chronic high fat or high carbohydrate diet when compared to animals fed a standard diet. If a minimum degree of or increase in vagal tone is required to feel satiated, then the reduction observed in this study would be expected to drive both groups to consume more and gain more weight, the latter of which was shown to be true. These results suggest VNS systems to reduce obesity will need to increase the change in vagal tone by approximately 4× in order to convey stomach distension. Further studies are needed to determine stimulation parameters required for this fourfold increase in neural signaling.

## Supplementary Information

Supplementary Information.

## Data Availability

The datasets generated and analyzed during the current study will be made available in the Harvard Dataverse repository upon acceptance of the manuscript.
