# Supplementary material for: Both high fat and high carbohydrate diets impair vagus nerve signaling of satiety
Source: Sci Rep. 2021 May 17;11:10394. doi: 10.1038/s41598-021-89465-0 (PMC8128917; doi:10.1038/s41598-021-89465-0)
Supplement: Supplementary file 1 — Supplementary Information. [file 41598_2021_89465_MOESM1_ESM.docx]

**Both High Fat and High Carbohydrate Diets Impair Vagus Nerve Signaling of Satiety**

Hailley Loper^1,2^, Monique Leinen^1,2^, Logan Bassoff^1,2^, Jack Sample^3,4^, Mario Romero-Ortega^5^, Kenneth J. Gustafson^3,6^, Dawn M. Taylor^3,6,7^, Matthew A. Schiefer^1,3,6^

**Supplement**

*Additional Details for Figure 1*

Supplementary Table 1: Vitals Metrics (μ±SEM)

| **Metric** | **S** | **LF-HC** | **HF-LC** | **p** |
| --- | --- | --- | --- | --- |
| Age (days) | 387±42 | 343±31 | 353±36 | 0.691 |
| Weight (g) | 620±16 | 689±36 | 720±37 | 0.106 |
| BMI (g/cm^2^) | 0.97±0.04 | 1.03±0.04 | 1.11±0.05 | 0.055 |
| Abd. Circum. (cm) | 24.4±0.4 | 25.9±0.7 | 26.8±0.9 | 0.078 |
| Isoflurane (%) | 1.8±0.1 | 1.6±0.1 | 1.9±0.1 | 0.049 |
| Heart Rate (bpm) | 249±9 | 283±13 | 294±10 | 0.017 |
| Breathing Rate (bpm) | 45±2 | 48±3 | 52±1 | 0.123 |
| SpO_2_ (%) | 99.1±0.1 | 98.6±0.5 | 98.7±0.3 | 0.532 |
| Max Distension Volume (mL) | 5.3±0.2 | 5.4±0.2 | 5.0±0.1 | 0.182 |

*Additional Controls*

Other control experiments were conducted in five recently deceased animals to verify the distension process itself did not create artifacts that would contribute to the measured neural metrics. Euthanasia in these animals had occurred several minutes to a few hours prior to distension. In the 12 trials that were conducted in five recently euthanized rats, stomach distension did not result in a percent change in the AUC of the vagal recording that significantly differed from baseline (p=0.374) (Supplemental Figure 1). The percent increase in AUC during distension was significantly less in this group than in living rats (p=0.005). In fact, the percent change in AUC tended to decrease over time due to a progressive decrease in tone following euthanasia. Although time and distension could not be separated, there was not an observable increase in AUC associated with individual distensions.

**Supplemental Figures**


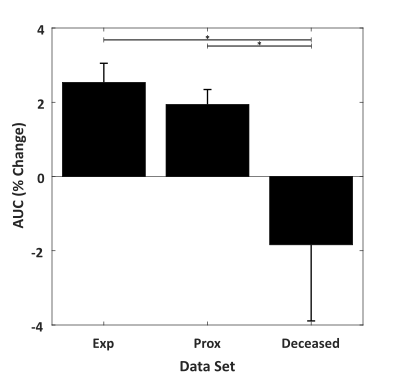


**Supplementary Figure 1:** Comparison of the experimental group (Exp) versus two of the control groups: the groups with a proximal vagal nerve transection (Prox) and the group that had been euthanized prior to the experiment (Deceased). Significant differences (p<=0.05) are denoted with “*”.


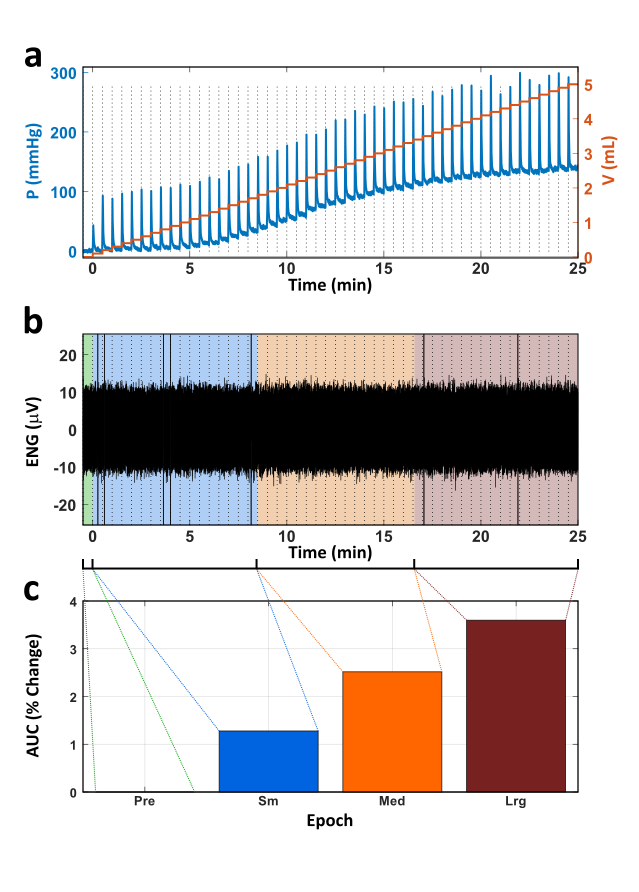


**Supplementary Figure 2:** Example of defining dynamic epochs in a trial with a distension step size of 0.1 mL. Data from animal on the HF-LC diet.


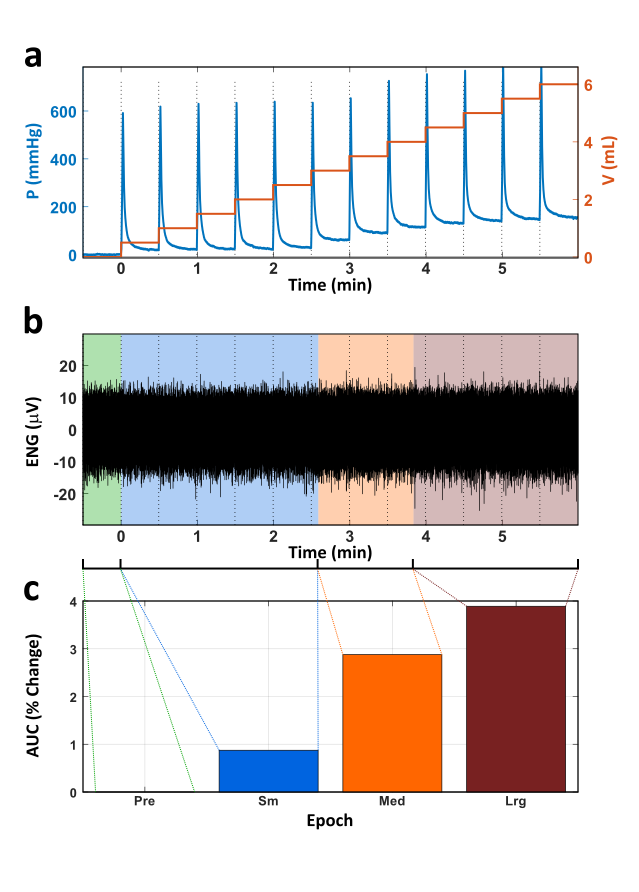


**Supplementary Figure 3:** Example of defining dynamic epochs in a trial with a distension step size of 0.5 mL. Data from animal on the LF-HC diet.


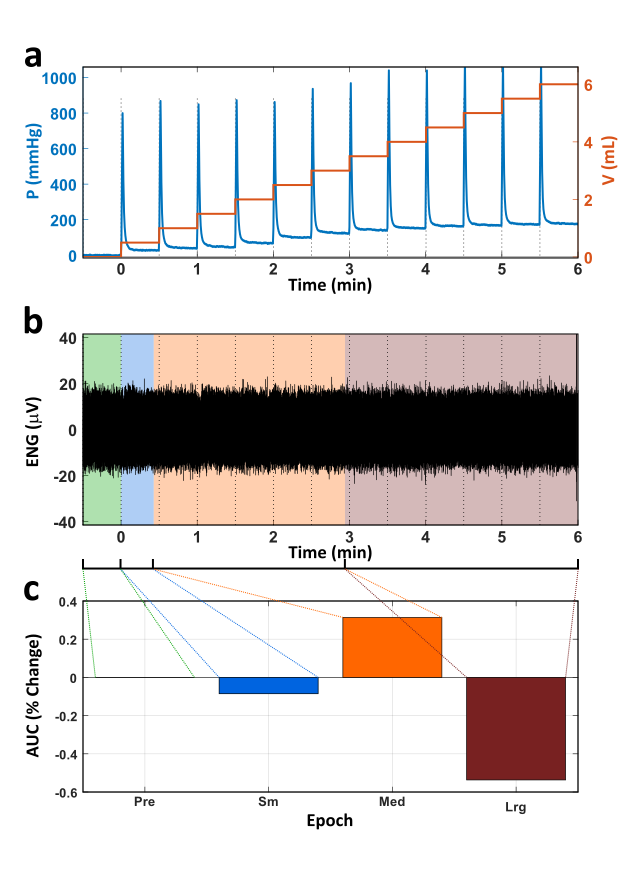


**Supplementary Figure 4:** Example of defining dynamic epochs in a trial with a distension step size of 0.5 mL. Data from a deceased control animal.
